# Transition metal pincer catalysts for formic acid dehydrogenation: a mechanistic perspective

**DOI:** 10.3389/fchem.2024.1452408

**Published:** 2024-08-26

**Authors:** N. Sai Kumar, Anubendu Adhikary

**Affiliations:** School of Advanced Sciences, VIT-AP University, Amaravati, Andhra Pradesh, India

**Keywords:** formic acid dehydrogenation, pincer complexes, ligand cooperativity, decarboxylation, transition metal hydride, transition metal formate

## Abstract

The storage and transportation of hydrogen gas, a non-polluting alternative to carbon-based fuels, have always been challenging due to its extreme flammability. In this regard, formic acid (FA) is a promising liquid organic hydrogen carrier (LOHC), and over the past decades, significant progress has been made in dehydrogenating FA through transition metal catalysis. In this review, our goal is to provide a detailed insight into the existing processes to expose various mechanistic challenges associated with FA dehydrogenation (FAD). Specifically, methodologies catalyzed by pincer-ligated metal complexes were chosen. Pincer ligands are preferred as they provide structural rigidity to the complexes, making the isolation and analysis of reaction intermediates less challenging and consequently providing a better mechanistic understanding. In this perspective, the catalytic activity of the reported pincer complexes in FAD was overviewed, and more importantly, the catalytic cycles were examined in detail. Further attention was given to the structural modifications, role of additives, reaction medium, and their crucial effects on the outcome.

## Graphical Abstract

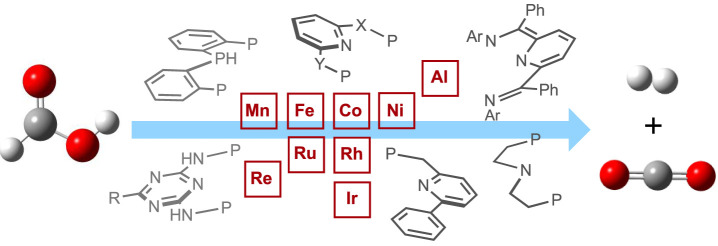

## 1 Introduction

**SCHEME 1:**
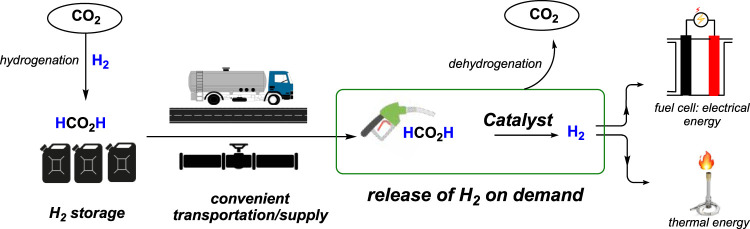
Formic acid as a hydrogen carrier for CO_2_ hydrogenation/dehydrogenation.

The demand for dihydrogen (H_2_) as an alternative to carbon-based fossil fuels is growing enormously as it is renewable and produces clean energy. However, because of its gaseous state and high flammability, the storage and transportation of H_2_ have always been challenging (Schlapbach and Züttel, 2001; Turner, 2004; Felderhoff et al., 2007; Schüth, 2009; Myers and Berben, 2014; Zhu and Xu, 2015; He et al., 2016; Lu et al., 2016). Compared to current storage techniques such as compression, liquefaction, or adsorption, storing H_2_ chemically through liquid organic hydrogen carriers (LOHCs) is far safer and more convenient. In this regard, formic acid (FA) is a promising candidate due to its high volumetric capacity of H_2_ (53.4 g/L at STP), non-flammability, and non-toxicity (Grasemann and Laurenczy, 2012). For utilizing and implementing formic acid to store and transport H_2_, two chemical processes must be efficient and economically viable: a) formic acid production and b) H_2_ generation from FA. Current industrial processes for synthesize FA through its long-known straightforward procedures using carbon monoxide are inexpensive. Most importantly, convenient FA production methods from the hydrogenation of CO_2_ have been achieved through transition metal catalysis (Wang et al., 2015; Álvarez et al., 2017; Yin et al., 2019). Therefore, combining H_2_ with CO_2_ to obtain formic acid, followed by its decomposition to release the H_2_, provides a viable, carbon-neutral solution for using formic acid as an equivalent to liquid hydrogen (Scheme 1).

Studying the convenience and applicability of various LOHCs has been a burgeoning area of research, particularly in the automobile industry. Along this line, at the Eindhoven University of Technology in the Netherlands, the world’s first bus powered by formic acid has been developed. This breakthrough prototype by the startup “Team Fast” (Formic Acid Sustainable Transportation) has shown how safe, more efficient, and a carbon-neutral fuel formic acid can be (http://teamfast.nl, http://www.dens.one). In the past decades, several catalytic systems, under both homogeneous and heterogeneous conditions, have been studied and applied for the dehydrogenation of formic acid (Grasemann and Laurenczy, 2012; Andérez-Fernández et al., 2016; Mellmann et al., 2016; Singh et al., 2016; Bernskoetter and Hazari, 2017; Li and Xu, 2017; Algi et al., 2018; Katerina et al., 2018; Onishi et al., 2022; Wei et al., 2022). Although these catalysts have significantly contributed to the understanding and advancement of the formic acid dehydrogenation (FAD) process, most reported methodologies are associated with one or more limitations: a) generation of CO-contaminated H_2_; b) short-lived and non-reusable catalysts; c) costly and complicated synthetic procedures of the catalyst; and most commonly, d) use of additives and e) costly or organic reaction medium. To overcome these challenges, it is essential not only to develop a new generation of catalysts but also to study and understand the details of existing procedures. In this review, our primary focus is to understand and analyze various mechanistic pathways involved in metal-catalyzed FAD.

**FIGURE 1:**
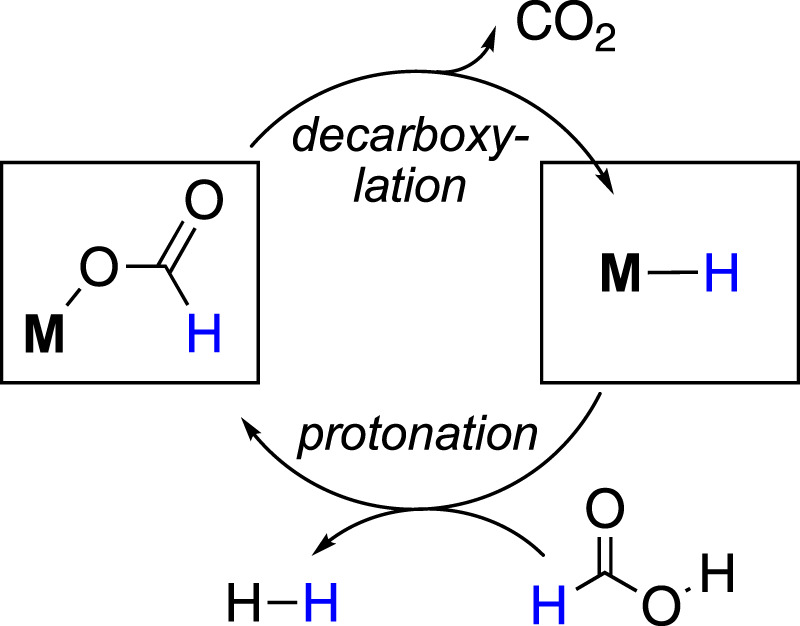
Formic acid dehydrogenation cycle involving metal–hydride and metal–formate complexes.

**SCHEME 2:**
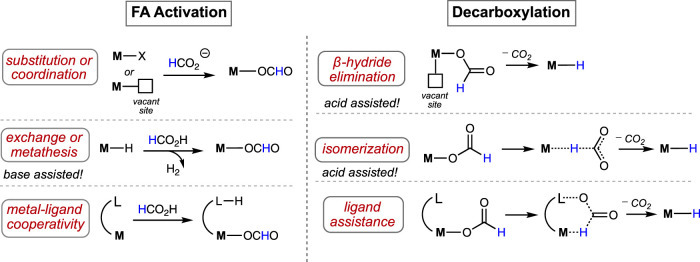
Various pathways to activate formic acid (left) and decarboxylate the metal–formate complex (right).

In general, the formic acid decomposition pathway can be divided into two major parts: (a) formation of H_2_ and (b) release of CO_2_. Among most of the existing catalytic cycles, it is noted that two intermediates play the major role: metal-bound hydride (M-H) and metal-bound formate (M-OCHO) (Mellmann et al., 2016; Bernskoetter and Hazari, 2017; Algi et al., 2018; Katerina et al., 2018; Onishi et al., 2022; Wei et al., 2022). In the dihydrogen formation step, which can also be broadly termed “protonation” or “FA activation,” the M-H reacts with formic acid, producing H_2_ and metal formate (Figure 1). In another step, the formate complex undergoes CO_2_ elimination, regenerating the M-H. In principle, it appears as a straightforward cycle; however, a number of factors may critically be involved in both steps, which brings up numerous possibilities in the course of the reaction (Scheme 2). Based on the catalyst structure, properties, and reaction conditions, various options in FA activation are possible, which include traditional coordination, acid–base-type interaction, ligand participation, or oxidative addition (less likely). Similarly, the decarboxylation pathways may follow classical *β*-hydride elimination, isomerization followed by CO_2_ release, or through metal–ligand cooperation. Furthermore, these processes were found to be influenced by different additives, such as salts, bases, or Lewis acids. Unfortunately, elucidation of the operating mechanism of a particular process is often challenging due to the high reactivities of transient intermediates. Hence, for a comprehensive understanding of these mechanisms, those catalytic cycles will be preferred where intermediates are isolable or can be characterized *in situ*. In this context, the pincer ligands are well known for providing stability to plenty of metal complexes through stronger binding (Koten and Milstein, 2012; Szabó and Wendt, 2014; Morales-Morales, 2018; Piccirilli et al., 2020). Thus, we became particularly interested in examining catalysts with rigid pincer backbones, expecting to acquire a finer mechanistic insight. In addition, these ligands allow us to tune the reactivity of the metal center by accommodating different substituents with flexible electronic and steric properties, which will provide additional findings about the processes. In the last 15 years, metal complexes ligated with tridentate pincers have shown a tremendous contribution in dehydrogenating formic acid (Table 1, **1A**–**24A**). In this report, we presented these catalysts collectively and compared their activity in FAD. More importantly, a thorough inspection of the reaction mechanisms was conducted, and the effects of essential factors such as backbone structure, ancillary ligands, various substituents, and additives were discussed in detail. The connection between strategic modifications and their consequences was also illustrated.

**TABLE 1:** Pincer catalysts for formic acid dehydrogenation.

Catalyst ^ref.^		Reagent^a^	Condition^b^	TON (time)^c^	TOF h^-1^ (time)^c^
**1A** (Filonenko et al., 2014)	[(^tBu^PNP)**Ru** ^II^(CO)H_2_]	FA/Et_3_N	90°C, DMF	326,500 (2 h)	257,000 (1 h)
		FA/DBU	90°C, DMF	310,000 (5.5 h)	93,100 (1 h)
		FA/N-Hex	90°C, DMF	706,500 (4.3 h)	256,000 (1 h)
**2A** (Kothandaraman et al., 2015)	[(^Ph^PNP)**Ru** ^II^(Cl) (BH_4_)H]	FA/NaOH	70°C, dioxane–H_2_O	994 (1.5 h)	820 (1 h)
**3Aʹʹ** (Anderson et al., 2017)	[(^tBu^PONOP)**Ru** ^II^(OTf)H]	FA/Et_3_N	80°C, C_6_H_5_Cl	190,000 (24 h)	7,916 (24 h)
**4A** (Pan et al., 2016)	[(^tBu^PN_3_P)**Ru** ^II^(CO)H]	FA	50°C, DMSO	95,000 (40 h)	2,380 (40 h)
		FA/Et_3_N	50°C, DMSO	250,000 (100 h)	2,500 (100 h)
		FA/Et_3_N	90°C, DMSO	1,100,000 (150 h)	7,333 (150 h)
**5A** (Agapova et al., 2019)	[(^iPr^PNP)**Ru** ^II^(Cl) (CO)H]	FA	80°C, BMIM OAc	16,750 (18 h)	930 (18 h)
**5A** (Agapova et al., 2019)	[(^iPr^PNP)**Ru** ^II^(Cl) (CO)H]	FA/KOH	92°C, triglyme-H_2_O	6,165 (3 h)	1,693 (1 h)
**5Aʹ** (Agapova et al., 2019)	[(^iPr^P^Me^NP)**Ru** ^II^(Cl) (CO)H]	FA/KOH	92°C, triglyme-H_2_O	7,800 (3 h)	6,492 (1 h)
**6A (** Kar et al., 2021)	[(^iPr^P^acr^NP)**Ru**(CO)H]	FA	95°C, neat	1,701,150 (1,244 h)	2,186 (45 min)
7A (Zell et al., 2013)	[(^tBu^PNP)**Fe** ^II^(CO) (H)_2_]	FA/Et_3_N	40°C, THF	2,000 (3 h)	836 (1 h)
**8A** (Bielinski et al., 2014)	[(^iPr^P^H^NP)**Fe** ^II^(OCHO) (CO)H]	FA/LiBF_4_	80°C, dioxane	983,642 (9.5 h)	196,728 (1 h)
**9A** (Mellone et al., 2016)	[(^iPr^PNNNP)**Fe** ^II^(CO)H_2_]	FA/Et_3_N	60°C, THF	1,000 (2 h)	770 (1 h)
**10A** (Pandey et al., 2022; Pandey et al., 2023a; Pandey et al., 2023b)	[(^iPr^P^H^PP)**Fe** ^II^(CO)H_2_]	FA	80°C, dioxane	859 (8 h)	473 (1 h)
**11A** (Tanaka et al., 2011)	[(^iPr^PNP)**Ir** ^III^H_3_]	FA/Et_3_N	80°C, ^*t*^BuOH	5,000 (4 h)	120,000 (1 min)
**12A (** Alrais et al., 2024)	[(^iPr^PNNNP)**Ir** ^III^H_3_]	FA/Et_3_N	60°C, THF	1,000 (2 h)	770 (1 h)
**13A** (Jongbloed et al., 2016)	[(^tBu^PNC)**Rh** ^I^(CO)]	FA	75°C, dioxane	140 (1 h)	169 (15 min)
**14A** (Zhou et al., 2019)	[(^Ph^P^H^NP)**Co** ^I^Cl]	FA/HCO_2_K	60°C, H_2_O	894 (3 h)	240 (20 min)
**15A (** Lentz et al., 2021)	[(^Cy^P^RN^PP)**Co** ^I^(CO)H]	FA	80°C, dioxane	200 (1 h)	-
**16A** (Osipova et al., 2023)	[(^tBu^PCP)**Pd** ^II^(OC)**Mo**(CO)_2_Cp]	FA	50°C, toluene	100 (30 min)	-
**17A (** Enthalere et al., 2014)	[(^tBu^PCP)**Ni** ^II^H]	FA/DMOA	80°C, PC	481 (2 h)	240 (2 h)
**18A** (Vogt et al., 2014)	[(^tBu^PNP)**Re** ^I^(CO)_2_]	FA	120°C, dioxane	2,600 (24 h)	108 (24 h)
**19A** (Tondreau and Boncella, 2016)	[(^iPr^P^H^NP)**Mn** ^I^(OCHO) (CO)_2_]	FA	65°C, dioxane	130 (14 h)	47 (1 h)
**20A** (Andérez-Fernández et al., 2016)	[(^iPr^P^H^NP)**Mn** ^I^(CO)_2_Br]	FA/DMOA	60°C, PC	283 (5 h)	57 (1 h)
**21A (** Anderson et al., 2017)	[(^tBu^PONNP)**Mn** ^I^(CO)_2_]Br	FA/Et_3_N	80°C, C_6_H_5_Cl	500 (336 s)	8,600 (80 s)
**22A** (Dutta et al., 2023)	[(^tBu^PNNNP)**Mn** ^I^(CO)_2_]Br	FA/Et_3_N	90°C, DMSO	15,200 (49 h)	2,086 (10 min)
**23A** (Wei et al., 2022a; Wei et al., 2022b)	[(^iPr^PN^RAr^NNP)**Mn** ^I^(CO)_2_]Br	FA/LysK	90°C, THF-H_2_O	27,600 (12 h)	2,300 (12 h)
**24A** (Myers and Berben, 2014; Lu et al., 2016)	[(^Ar^NNN)**Al** ^III^H]	FA/Et_3_N	65^o^C, THF	2,200 (1 h)	5,200 (15 min)

^a^ FA, formic acid; DBU, 1,8-diazabicyclo (5.4.0)undec-7-ene; DMOA, dimethyl octylamine; LysK, lysine^–^K^+^.

^b^ Dioxane, 1,4-dioxane; BMIM OAc, 1-butyl-3-methylimidazolium acetate; PC, propylene carbonate.

^c^ Turnover was calculated at (time).

## 2 Group VIII (Ru and Fe) metal complexes

### 2.1 Ruthenium

**FIGURE 2:**
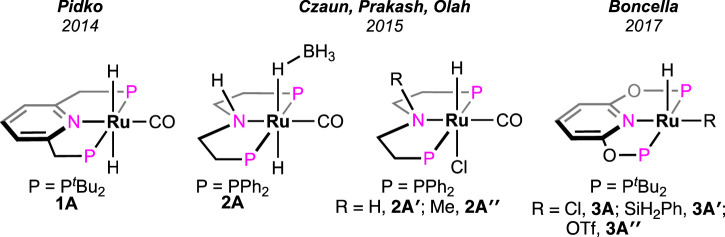
Ruthenium pincer catalysts for the dehydrogenation of FA with base additives.

**SCHEME 3:**
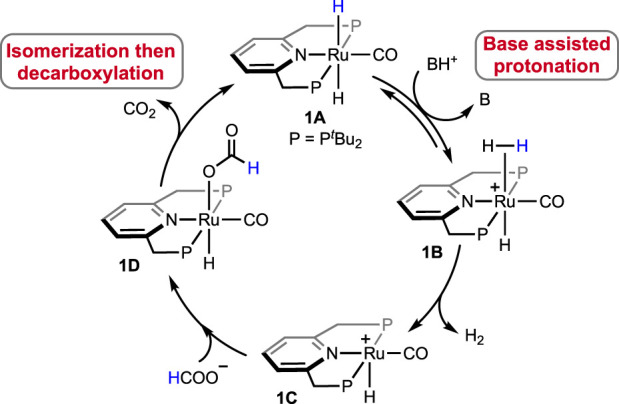
Proposed mechanism of (PNP)Ru-catalyzed FA dehydrogenation.

In 2014, a ruthenium complex with a PNP-ligand [PNP = 2,6-bis(di-*tert*butylphosphinomethyl) pyridine; Figure 2, **1A**] was applied in formic acid dehydrogenation by Filonenko et al. (2014). The catalytic reactions were set up in a batch reactor and studied under continuous FA addition. Over a range of temperatures (65–90°C), high turnovers by **1A** were obtained in the presence of a base such as triethylamine, trihexylamine, and 1,8-diazabicyclo (5.4.0)undec-7-ene (DBU). Among different polar solvents, DMF was found to be most effective considering the catalytic activity and their stability. However, no conversion was observed in the polar protic solvent, e.g., ethanol, possibly due to the insolubility of the catalyst. After a thorough experimental and computational study, a comprehensive mechanistic cycle was proposed, as shown in Scheme 3. In the first step, protonation of the metal-bound hydride resulted in the formation of H_2_. Afterward, its rapid dissociation generated a cationic Ru intermediate. The coordination of the formate ion to this intermediate produced Ru–formate, which was successfully isolated and characterized in a separate experiment. The release of CO_2_ from Ru–formate completed the cycle, regenerating **1A**. As noted, the base additives played an important role in the process, and interestingly, their proportion with respect to FA was found to be critical. It was observed that Et_3_N and DBU influenced the rate in an opposite way. In the case of triethylamine, the rate decreased with an increase in the acid-to-amine ratio, whereas, with DBU, the rate varied proportionally with the acid/amine concentration. This behavior was believed to be controlled by the strength of basicity and the reverse nature of the protonation step (Scheme 3, **1A**→**1B**). In the protonation step, DBU being a stronger base (pK_a_ = ∼11.5 in water), its conjugate acid is reluctant to protonate the ruthenium-bound hydride, making H_2_ formation a rate-limiting step. On the other hand, for Et_3_NH^+^ (pK_a_ = ∼10.5 in water), protonation of Ru-H is more energetically favorable, ensuing decarboxylation as the slower step (Scheme 3, **1D**→**1A**). This base-dependent switching of *rds* was further supported by the kinetic isotope effect study. The activation energy of the decarboxylation step, which is rate-limiting for the Et_3_N-assisted cycle, was calculated as 74 kJ mol^-1^.

Olah et al. investigated the catalytic activity of another ruthenium–pincer complex with a non-aromatic P^H^NP backbone (P^H^NP = HN{CH_2_CH_2_(PR_2_)}_2_; R = Ph; Figure 2, **2A**) (Kothandaraman et al., 2015). Using the complex, different formate salts were successfully dehydrogenated in a dioxane–water medium under convenient conditions. Lithium and sodium formates were more reactive in producing H_2_ than their potassium and cesium salts. A catalytic cycle similar to that shown in Scheme 3, involving Ru–formate and Ru-hydride as intermediates, was proposed. To uncover the presence of any ligand cooperativity, complexes having a methyl substituent on the central nitrogen were examined (complex **2A**″). Although pre-catalysts **2A**′ and **2A**″ were equally efficient in FAD, the possibility of ligand participation was not ruled out. Reaction kinetics and isotopic studies suggested the CO_2_ elimination step as rate-determining, whose activation energy was also calculated (80.7 kJ mol^-1^). In another report, Boncella et al. explored the reactivity of the (PONOP)-ruthenium catalyst, which was structurally similar to **1A** (PONOP = 2,6-bis(diisopropylphosphinito)pyridine; Figure 2, **3A**) (Anderson et al., 2017). These catalysts were found to be highly efficient in dehydrogenating an equimolar mixture of FA and Et_3_N, even under high pressure. For instance, at 80°C, a pressure of 10 atm was built up in 2 min, starting from 1.58 mL of reagents. A fast reaction with TOF of 36,000 h^-1^ was achieved; however, the catalyst was found to be ineffective without the amine.

**SCHEME 4:**
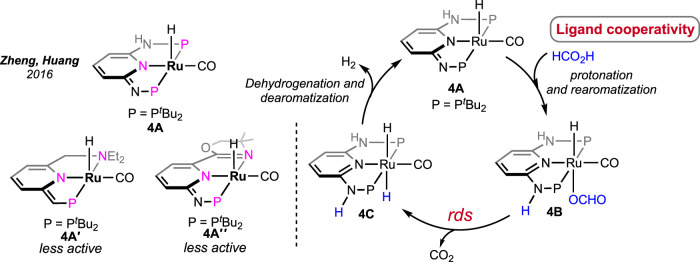
Ruthenium pincer catalysts for base-free FAD (left). Proposed mechanistic cycle involving ligand cooperation (right).

All the aforementioned Ru catalysts were air-sensitive and required external bases for effective dehydrogenation. Zheng and Huang et al. prepared an air- and moisture-stable (PN_3_P)-ligated ruthenium pincer (Scheme 4, **4A**) that displayed comparable reactivity but at a lower temperature (50°C) (Pan et al., 2016). Interestingly, the catalyst worked under base-free conditions at a high speed (TOF of 2,380 h^-1^). Furthermore, in the presence of a stoichiometric quantity of triethylamine, the TOF increased up to 2,500 h^-1^. An increase in efficiency was also attained at elevated temperatures (90°C; TOF of 7,333 h^-1^ and TON up to 1,100,000). This catalyst was successful in bypassing the requirement of an amine, suggesting internal assistance from the ligand. In an observation, the reaction between **4A** and one equivalent of formic acid instantly generated complex **4B** bearing a protonated N-center, which strongly supported the capability of imine nitrogen to perform as an internal base. Accordingly, in the base-free catalytic cycle, the activation of formic acid via ligand protonation was well established as a critical step (Scheme 4). Nevertheless, the deinsertion of CO_2_ from Ru-OCHO was determined as the slower step. In another study, complexes **4A**′ and **4A**″, having similar ligand frameworks, were examined; however, they displayed slower catalysis than **4A**, illustrating the influence of phosphine donors.

**FIGURE 3:**
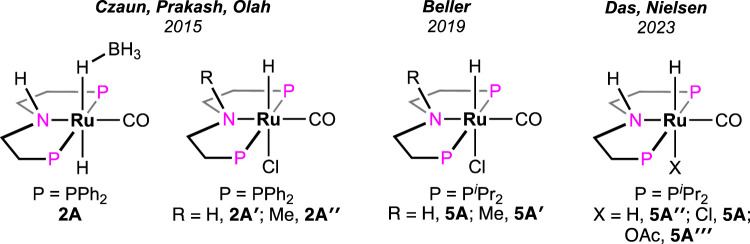
Bis(phosphine)-based non-aromatic (PNP) Ru pincer complexes applied in FAD.

**SCHEME 5:**
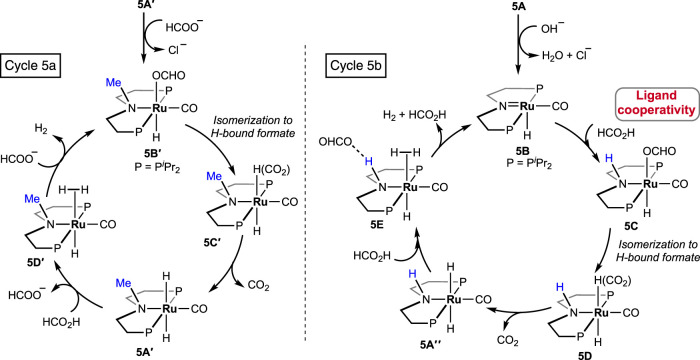
FAD cycles by catalysts **5Aʹ** (left) and **5A** (right).

In 2021, Beller et al. performed a comparative study between (P^H^NP)-Ru pincer complex **5A** and its N-methylated version **5A′** (P^Me^NP-Ru; P^Me^NP = MeN{CH_2_CH_2_(PR_2_)}_2_; R = ^*i*^Pr, Figure 3) (Agapova et al., 2019). Surprisingly, the complex with a methylated backbone exhibited a faster FA dehydrogenation irrespective of the reaction condition. In the case of (P^H^NP)-Ru pincer **5A**, the involvement of Ru-*N* in the catalytic cycle was expected, whereas for **5A′**, ligand participation is not possible, as shown in Scheme 5. The enhanced reactivity of **5A′** implied that ligand cooperativity was not advantageous. When the effect of pH on the reaction rate was tested, H_2_ generation was found to be faster under acidic conditions (pH 4.5). Although the rate was moderate at a pH value of 13, a more satisfactory conversion and rate were obtained when the reaction was carried out in a buffer solution (pH range: 4.5–8.5). Displaying a low-pH preference indicates the crucial role of the protonation step in the mechanism (cycle 5a, **5A′**→**5D′**; cycle 5b, **5A**″→**5E**). During the catalysis by the (P^Me^NP)Ru pincer, both the intermediates **5A′** and **5B′** were detected in the reaction mixture (cycle 5a). Possibly, the protonation step is highly influential in these cycles, and in the case of N-methylated ruthenium hydride, it is energetically more favorable. Activation energies for **5A** and **5A′**-catalyzed FAD were experimentally calculated as 85.9 kJ mol^-1^ and 66.9 kJ mol^-1^, respectively. A difference of ∼20 kJ mol^-1^ between their activation energies supports the no-benefit of ligand cooperativity on the overall rate. However, in an ionic liquid medium, complex **5A** and its derivatives were proven to be excellent catalysts for CO_2_ hydrogenation–dehydrogenation. Das and Nielsen et al. successfully applied complexes **2A**, **2A′**, **5A**, **5A**″, and **5A‴** in producing H_2_ from formic acid under additive-free conditions (Figure 3) (Piccirilli et al., 2023). Although similar turnovers were shown by all these catalysts, those having phenyl groups on *P*-donors (**2A** and **2A′**) exhibited higher stability over the *P*-isopropyl analogs (**5A**, **5A**″, and **5A‴**). The ionic liquid, 1-butyl-3-methylimidazolium *acetate* (BMIM OAc), acted not only as an effective medium but also played a crucial role in activating the catalyst. Spectroscopic investigation disclosed that irrespective of the applied (PNP)Ru complexes, the resting state during catalysis was the *acetate* complex **5A‴**. This observation confirmed that the acetate ion from the solvent was directly involved in triggering the catalytic activity.

**FIGURE 4:**
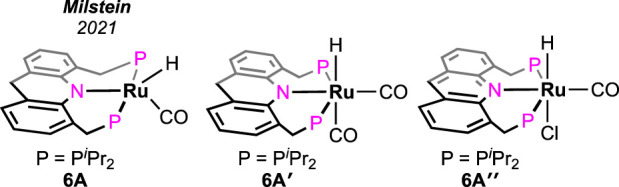
Acridine-based (PNP) ruthenium catalysts for solvent-less, additive-less FA decomposition.

**SCHEME 6:**
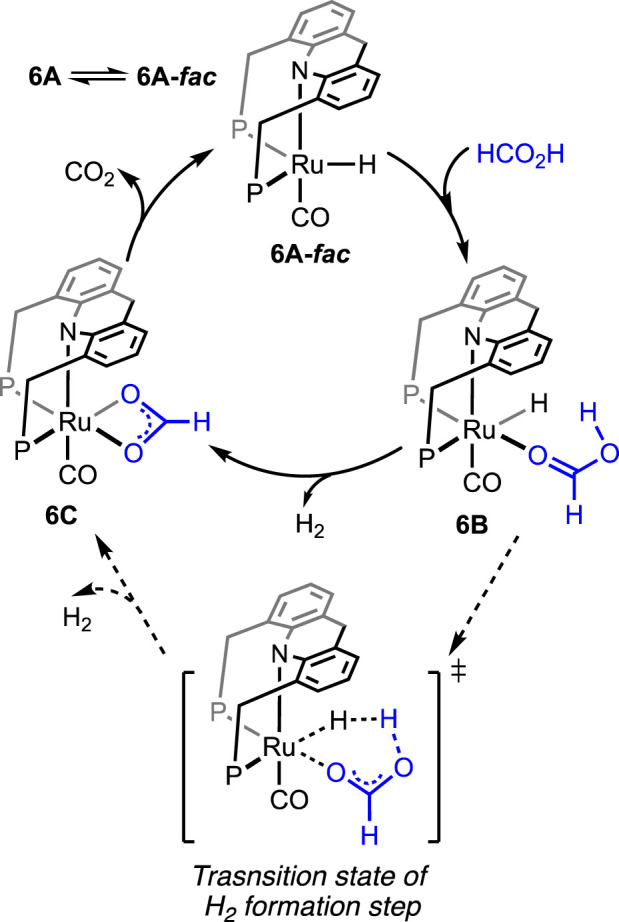
Proposed mechanistic cycle of **6A**-catalyzed dehydrogenation of FA.

Without using any solvent or additive, the catalytic dehydrogenation of formic acid was achieved by Kar et al. (2021). The ruthenium hydride complex supported on the 9H-acridine-based bis(phosphine) ligand has shown remarkable turnovers in producing H_2_ at 95°C (Figure 4, **6A**). More importantly, a pressure of 100 bar was attained starting from 5 mL of 85% formic acid. Experimental investigation revealed an initial binding of formic acid to the ruthenium center (Scheme 6, **6B**). The presence of the FA-coordinated ruthenium complex in the reaction mixture was confirmed using NMR spectroscopy. A detailed theoretical calculation showed a stabilizing hydrogen-bond interaction between the FA proton and Ru-H, which favored dihydrogen generation. Through a six-membered cyclic transition state, eventually, the formation of the ruthenium–formate complex was proposed (Scheme 6, **6B** → **6C**). Although the formate complex was not observed during catalysis, it was isolated from a separate experiment by reacting **6A** with excess CO_2_ (complex **6C**, κ^2^-coordination mode of HCO_2_^−^). As anticipated, under a CO_2_-free environment, decarboxylation from **6C** was observed, resulting in the regeneration of the hydride **6A**. Kinetic isotope effects and DFT calculation demonstrated the CO_2_ release as a slower process than the elimination of H_2_. Furthermore, the activation parameters were experimentally calculated as 17.01 kcal mol^-1^ (ΔH^≠^) and −20.36 cal mol^-1^ K^−1^ (ΔS^≠^). On an interesting note, even though the formation of CO was below 20 ppm, complex **6A′** was detected during the reaction, indicating an interference from the byproduct CO. However, complexes **6A′** and **6A**″ were observed to be catalytically slower than **6A**.

### 2.2 Iron

**FIGURE 5:**
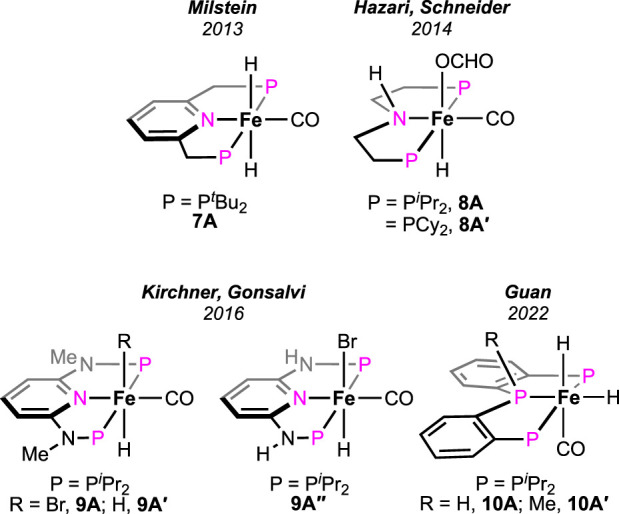
Different iron pincer complexes for FA decomposition.

**SCHEME 7:**
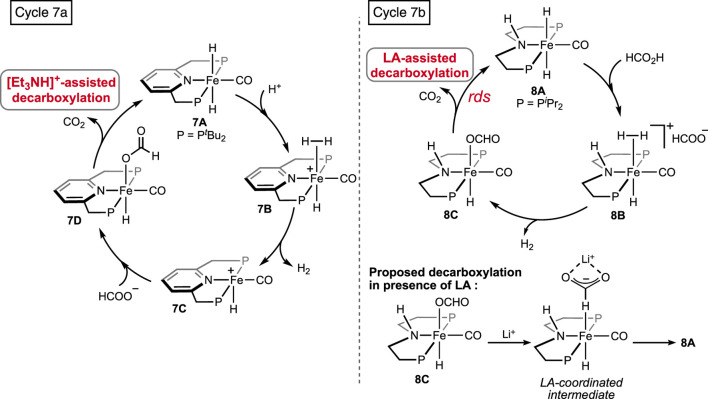
FAD catalytic cycles by iron pincers involving additive-assisted decarboxylation.

Supported by ligand frameworks similar to those used in Ru systems, a few iron complexes were synthesized and explored for formic acid decomposition. The (bis)phosphine-ligated iron hydride complex was first reported by Zell et al. (2013) (Figure 5, **7A**). Compared to the ruthenium analog (see **1A** in Figure 2), the iron hydride performed under a milder condition (40°C) but at a slower rate. In the presence of one equivalent of triethylamine, TON up to 2,000 with an initial TOF of 836 h^-1^ was obtained. The mechanistic cycle involved the usual protonation of the iron-bound hydride to produce H_2_ and its dissociation to generate a reactive cationic intermediate. Then, smooth coordination of formate to the iron center, followed by the release of CO_2_, completed the cycle (Scheme 7, cycle 7a). Although the Fe intermediates involved in this cycle are similar, as shown in Scheme 3, it is important to note the unusual role of the base additive. The amine has shown no effect on the rate of protonation (cycle 7a, **7A**→**7B**); rather, it was compelling to find experimentally that Et_3_NH^+^ facilitated the CO_2_ elimination process. As hypothesized by the group, a stabilizing interaction between the metal formate and the Bronsted acid (Et_3_NH^+^) sped up the decarboxylation. In addition, a classical *β*-hydride elimination of Fe-OCHO cannot be followed due to the unavailability of a vacant coordination site around the Fe(II) center; therefore, switching in the coordination mode of formate prior to CO_2_ release was necessary. As expected, DFT calculations strongly supported an intramolecular isomerization of iron-bound formate to carboxylate (Fe--H--CO_2_) in order to release the CO_2_.

Later, additive-promoted decarboxylation of iron–formate was well proven by Schneider and Hazari et al. (Bielinski et al., 2014; Zhang et al., 2015; Curley et al., 2018; Curley et al., 2021). The specialty of their finding was the role of the Lewis acid (LA) as a co-catalyst in FA dehydrogenation. Applying a (P^H^NP)–iron formate **8A-A′** as catalyst with 10 mol% Lewis acid, high conversion and turnover were accomplished (Figure 5; Scheme 7, cycle 7b). As evidenced, in the presence of Lewis acids, the decarboxylation from iron–formate was accelerated, which also suggested an energetically favorable interaction between the two. Among various LA additives, LiBF_4_ was found to be most effective in facilitating the deinsertion of CO_2_. As shown in Scheme 7, coordination of the formate with the lithium ion was proposed to be the key factor in lowering the energy barrier. In a comparison of potency between the Lewis acid and Bronsted acid (LiBF_4_ vs [Et_3_NH]^+^), a faster CO_2_ formation was observed for the triethylammonium ion under 0.1 mol% catalyst loading. However, in the case of LiBF_4_, interestingly, a higher rate and conversion were achieved with a lower catalyst concentration (0.001%). Irrespective of the additives used, during catalysis, iron–formate (**8c**) was the only observed species in the reaction mixture, which suggested its decarboxylation as the rate-determining step.

**SCHEME 8:**
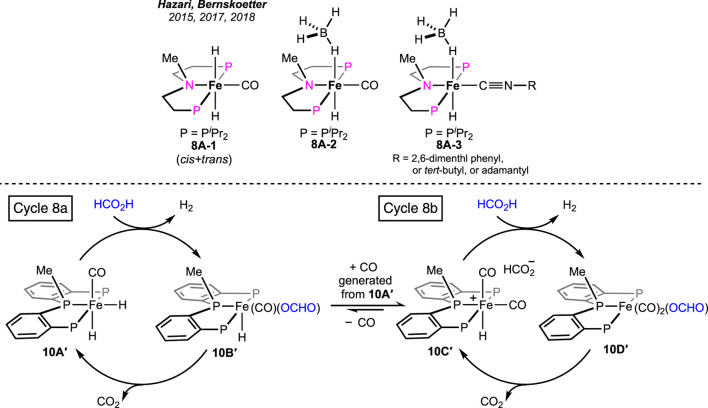
Tertiary amine-based (PNP)iron hydrides (above). Parallel catalytic cycles in FAD by (PPP)iron pincer complexes (stereochemistry is not confirmed for all the intermediates) (below).

In addition to FA dehydrogenation, the group extensively studied its reverse process, i.e., CO_2_ hydrogenation. By substituting the hydrogen atom on the *N*-donor with a methyl group (Scheme 8, **8A-1**), a huge improvement in the catalytic efficiency for CO_2_ hydrogenation was observed (Zhang et al., 2015). However, the corresponding effect on FA dehydrogenation was not reported. The group further studied the influences of the ancillary ligands by replacing the iron-bound CO with an isonitrile moiety. Prepared iron complexes **8A-2** and **8A-3** were found to be less efficient in catalytic FAD (Scheme 8). The presence of isonitrile functionality was proposed to destabilize the iron intermediates involved in catalysis, leading to catalyst decomposition. Later, it was discovered that the presence of small-size phosphines, such as PMe_3_ was useful in suppressing catalyst degradation (Curley et al., 2018; Curley et al., 2021).

Pyridine-based (PNNNP)-iron pincer catalysts showed a comparable efficiency with structurally similar **7A**, as reported by Gonsalvi and Kirchner (Figure 5, **9A**-**A′**) (Mellone et al., 2016). Amines were an optimum need to carry out the transformation. A similar catalytic pathway as in cycle 7a was proposed; however, the role of amine was found to be dissimilar. Experimentally, it was noted that the amine was involved in promoting the protonation step in contrast to favoring the decarboxylation, as observed in the case of the **7A**-catalyzed process. The presence of other Lewis acids, such as LiBF_4_, did not show any acceleration on the turnovers, implying no favor in the CO_2_ elimination step. In line with these experimental observations, computational calculations revealed the initial coordination of FA to the catalyst, followed by amine-led proton abstraction, resulting in the corresponding iron–formate, which further supported the positive impact of the amine on the protonation step. However, a point to note is that DBU, a stronger base, was less effective than triethylamine during catalysis. On another interesting note, poor activity was observed with complex **9A**″, despite being structurally eligible for metal–ligand cooperativity (Figure 5). Then, in 2022, novel (PPP)–iron dihydride complexes were investigated by Guan and Pandey [(*o*-*i*Pr_2_PC_6_H_4_)_2_PR (R = H or Me); Figure 5, **10A-A′**] (Pandey et al., 2022; Pandey et al., 2023a; Pandey et al., 2023b). Additive-less FAD was accomplished under convenient conditions using these catalysts. However, slow ligand dissociation, followed by decomposition of the catalyst, was observed, along with carbon monoxide formation. An extensive investigation led to a complicated reaction mechanism involving two parallel catalytic cycles (Scheme 8). Initial protonation of Fe-*cis*-(H)_2_, **10A′**, resulted in a *trans*-hydridoformate complex (cycle 8a, **10B′**), which was found to be in equilibrium with the iron–dicarbonyl complex (**10C′**), the proposed entry point to another cycle (cycle 8b). Nevertheless, as revealed experimentally, the rate-limiting step was the elimination of CO_2_ from iron–formate. No significant difference in the catalytic turnovers between **10A** and **10A′** was noted; thus, the possibility of ligand assistance during catalysis can be ruled out.

## 3 Group IX (Ir, Rh, and Co) metal complexes

### 3.1 Iridium

**FIGURE 6:**
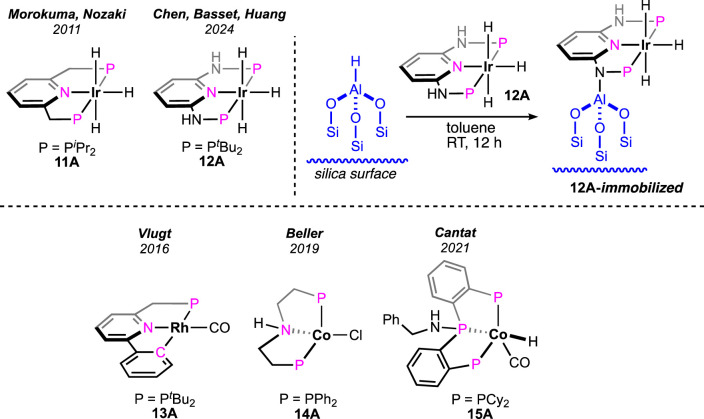
Iridium pincer complexes studied in FAD and the (^tBu^P^H^NN^H^NP)Ir trihydride immobilized on silica (above). Rhodium and cobalt pincer complexes studied in FAD (below).

Morokuma and Nozaki et al. studied the catalytic dehydrogenation of formic acid using a (^iPr^PNP)-ligated iridium(III) trihydride pincer (Figure 6, **11A**) (Tanaka et al., 2011). Without an amine additive, the catalyst showed a low conversion rate (18%) at 60°C. However, the catalytic performance significantly increased in the presence of triethylamine. At 80°C, quantitative conversion to H_2_ and CO_2_ was achieved within 4 h, displaying a high turnover frequency (120,000 h^-1^). Although no mechanistic investigation was performed for this FA dehydrogenation, the group looked into the details of CO_2_ hydrogenation, the reverse process of FAD. The participation of the ligand in the catalytic cycle was successfully demonstrated by experimental observations and DFT calculations. Following the principle of microscopic reversibility, these lines of evidence recommend ligand involvement in the FA dehydrogenation process. However, no further experiments were conducted to support the metal–ligand cooperativity. A recent advancement in iridium catalysis was made by Huang et al., where the formic acid dehydrogenation took place without any solvent. From a mixture of formic acid and cesium formate, dihydrogen was successfully produced by the (^tBu^P^H^NN^H^NP)iridium trihydride **12A** (Figure 6) (Alrais et al., 2024). Under the reaction condition (80–90°C), the cesium salt not only played the role of a base but also served as a potent medium. Fascinatingly, the group immobilized this highly active catalyst on an aluminum-bound fibrous silica nanosphere, which exhibited a remarkable upgrade in turnovers. As noted, the immobilized catalyst was two times more efficient in generating dihydrogen (TON 260,000 vs 540,000) (Figure 6). The catalytic pathway involved the formation of a metal formate from the reaction between FA and the catalyst, followed by its decarboxylation. Interestingly, after carrying out the transformation in an aqueous medium, an important role of water was found in stabilizing the reaction intermediates through hydrogen bond interactions. The detailed theoretical study further revealed the participation of ligand N-H and H_2_O molecules in the formic acid activation step. In an explanation of the higher reactivity of the immobilized catalyst, DFT calculations showed a greater negative charge density on the iridium of immobilized **12A**, which stimulated its protonation by FA.

### 3.2 Rhodium

**SCHEME 9:**
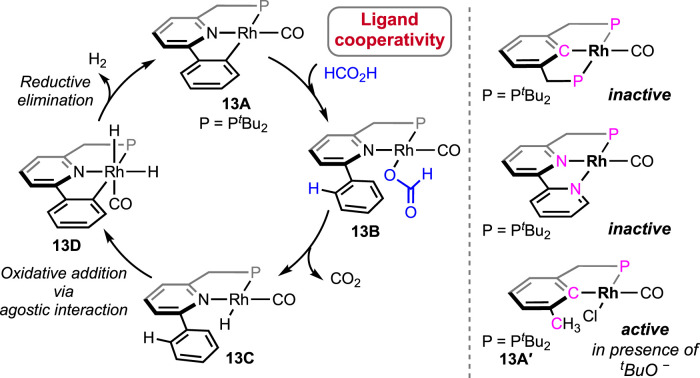
Mechanistic cycle of (^tBu^PNC)Rh(CO)-catalyzed FAD (left). Analogous rhodium pincer complexes attempted in FAD (right).

A rare type of metal–ligand cooperativity in activating formic acid was reported by van der Vlugt et al. (Figure 6) (Jongbloed et al., 2016). The rhodium(I) complex (**13A**), bearing a phenyl group as the pincer arm, satisfactorily decomposed formic acid under base-free conditions. Although a lower turnover than Ru and Fe catalysts was displayed, unlike previous cycles, the catalysis took an interesting route, which made it mechanistically valuable. The cycle started with the expected reaction between 13A and formic acid following a different path. The attached phenyl arm of **13A** abstracted H^+^, resulting in the opening of the metallacycle and the formation of a rhodium–formate bond (Scheme 9, **13B**). Then, the intermediate **13B** underwent decarboxylation, generating Rh(I)-hydride (**13C**). Oxidative addition of ^sp2^C-H to Rh(I) led to Rh(III)-dihydride, and finally, the reductive elimination of H_2_ completed the cycle. The use of HCO_2_D as the starting reagent resulted in deuteriation at the *ortho*-positions of the phenyl ring, which strongly supported the reversible cyclometallation pathway, as shown in Scheme 9. The computational study confirmed that Rh-OCHO, **13B**, was the most stable intermediate and the CO_2_ release was the slowest step. Evidently, the phenyl arm of the pincer ligand acted as an internal base, and as expected, no effect of external bases was noticed on the reaction rate. Furthermore, related complexes where carbanionic ligands were absent, such as (^tBu^PCP)- and (^tBu^PNN)-Rh(CO), were found completely inactive (Scheme 9). Another complex having a methyl arm, **13A**′, appeared as an active catalyst in the presence of a strong proton acceptor, *tert*-butoxide, emphasizing the crucial participation of the carbanionic ligand in the process. Despite these convincing lines of evidence, although less likely, mechanisms involving oxidative addition of FA to the Rh(I) center cannot be excluded.

### 3.3 Cobalt

**SCHEME 10:**
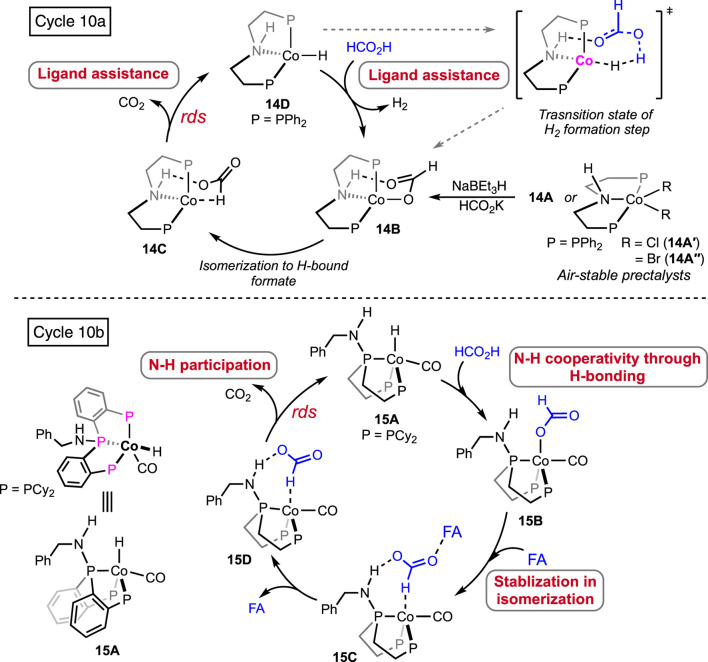
Proposed catalytic cycle of FA dehydrogenation by cobalt pincer **14A** (above, cycle 10a). DFT calculated catalytic pathway by cobalt pincer **15A** (below, cycle 10b).

(PNP)Pincer-bound cobalt complexes were investigated as pre-catalysts by Beller et al. (Figure 6, **14A**; Scheme 10, **14A′-A′′**) (Zhou et al., 2019). Looking at various pincer catalysts discussed earlier, the presence of a metal-bound strongly anionic group such as the hydride or aryl (in **13A**) was found in common. As obvious from the proposed catalytic cycles, these ancillary anionic ligands are critically involved in initializing the cycle by abstracting the proton from FA. On the other hand, the metal–formate can also be a starting point for these catalytic cycles. In this particular case, the cobalt halides were the catalyst precursor, and their activation required the formate salts. Nucleophilic displacement of the cobalt-bound chloride by HCO_2_^−^-generated cobalt–formate, which was found to initiate the dehydrogenation cycle (Scheme 10, cycle 10a). Isomerization of **14B** to **14C** occurred, followed by the CO_2_ deinsertion. Ultimately, proton transfer in the next step, generating H_2_, completed the mechanistic cycle. Interestingly, the critical involvement of pincer N-H to stabilize the intermediates through hydrogen bonding was unveiled by a detailed computational study. It is to be noted that possessing the same ligand, ruthenium, and iron pincers (see Figures 2, 5, **2A** and **8A**-**A**′, respectively) did not show any such N-H assistance or involvement in the catalysis. We believe that the tetrahedral configuration of the cobalt allowed an optimum fit to attain a geometrically stable transition state, which might be energy-demanding for octahedral pincers (for Ru and Fe). The results from kinetic isotopic studies strongly suggested the decarboxylation step as rate-limiting. In the improvement of aqueous-medium, air-stable cobalt dihalides, **14A**′-**A**″, showed satisfactory conversions. However, to activate these precursors, sodium triethyl borohydride was applied. Then, in 2021, an intelligent ligand design was reported by Cantat et al., accomplishing a co-catalyzed base-free FAD method (Lentz et al., 2021). The closer proximity of N-H to the metal center was attained after adding an amine functionality to the central *P*-donor of the cobalt (PPP)-pincer (Figure 6, **15A**). This structural refinement, as a consequence, successfully ruled out the need for an external base, as shown in cycle 10b (Scheme 10). A DFT study showed distinct N-H participation in the rate-limiting step, and hydrogen bond interactions were firmly proposed to stabilize the intermediates. In general, the FAD pathway involves polar intermediates and transition states, which makes the polar medium more suitable for carrying out the transformation. However, in this case, the efficiency of catalysts was found to be independent of the solvent polarity. This is an uncommon observation, and possibly, the stabilizing H-bond interactions present in the intermediates were strong enough to overcome the necessity of a polar environment.

## 4 Group X (Pd and Ni) metal complexes

### 4.1 Palladium

**FIGURE 7:**
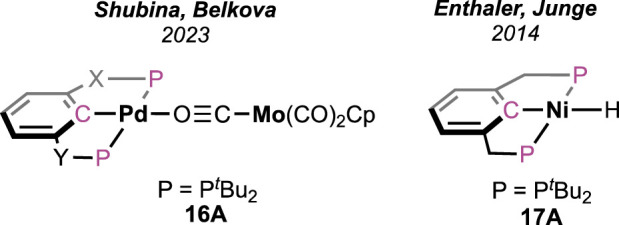
Palladium and nickel pincer complexes for FAD.

**SCHEME 11:**
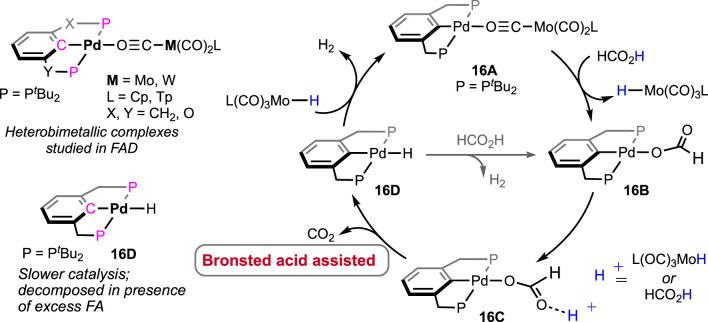
Various heterobimetallic complexes of palladium and molybdenum/tungsten in FAD (left) and the proposed catalytic cycle (right).

Shubina and Belkova et al. developed base-free FAD under milder temperatures (25–50°C) using palladium-based heterobimetallic catalysts (Figure 7; Scheme 11) (Osipova et al., 2023). These complexes behaved as a “transition metal analog of frustrated Lewis pairs.” Among the series of bimetallic complexes, **16A** was found to be the most efficient (Figure 7). Collaborative action from both metals was observed, where palladium acted as the acidic half and molybdenum as the basic counterpart. Systematic experimental investigation and evidence from infrared spectroscopy have led to a comprehensive mechanistic cycle (Scheme 11). At first, the proton was abstracted by the anionic counterpart [Mo(CO)_3_Cp]^−^, and the simultaneous coordination of HCO_2_^−^ ended up forming Pd-OCHO (**16A–16B**), resulting in the dissociation of the metal pair. Next, with the help of the Bronsted acid H-Mo or another molecule of HCO_2_H, the Pd–formate underwent decarboxylation, generating the hydride **16D**. Subsequently, in the last step, Pd-H and H-Mo combined, releasing dihydrogen. A spectral study, along with independent experiments, demonstrated a Pd-OCHO⋅⋅⋅⋅H^δ+^ hydrogen bond interaction, boosting the deinsertion of CO_2_. As shown in the catalytic cycle, the molybdenum unit plays a dual role: (a) the anion, i.e., [Mo(CO)_3_Cp]^−^, acts as the proton acceptor and (b) the conjugate acid, [Mo(CO)_3_Cp]H, actively participates in decarboxylation. Under the additive-free conditions, the use of bi(phosphinite)palladium-only complex **16D**, unfortunately, showed poor reactivity (Scheme 11). Moreover, slow decomposition of **16D** was noted, forming Pd(0) particles. These findings indicated the importance of metal–metal cooperativity not only in the catalysis but also in balancing the catalyst stability. Interestingly, toluene was used to perform all the studies, and no other solvent was tried, which might suggest the inessentiality of a polar medium to conduct dehydrogenation.

### 4.2 Nickel

Similar to complex **16D**, a bis(phosphonite)-ligated nickel hydride complex (Figure 7, **17A**) was investigated by Enthaler and Junge but in the presence of triethylamine (Enthalere et al., 2014). As expected, without amine, no reaction was observed, which verifies the role of an internal or external base in initializing the process. The best catalytic performance was observed in the propylene carbonate medium with dimethyl-*n*-octylamine as an additive. The formation of the intermediate Ni-OCHO and its decarboxylation are proposed to be the two major steps.

## 5 Group VI (Re and Mn) metal complexes

### 5.1 Rhenium

**SCHEME 12:**
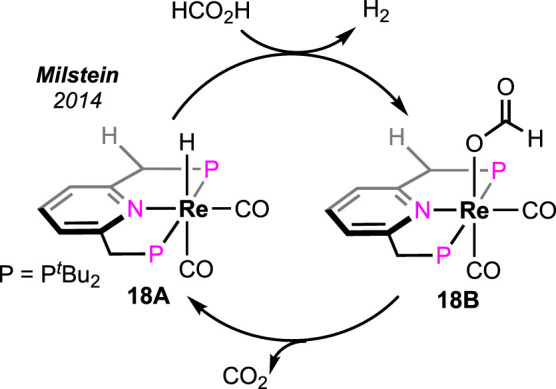
Base-free formic acid dehydrogenation by the rhenium hydride complex.

Milstein et al. intensely studied the pyridine-based (PNP)-rhenium complex in base-free FAD (Scheme 12) (Vogt et al., 2014). A low loading (0.03%) of the rhenium pincer successfully generated CO-free H_2_ at 180°C. When the reaction was carried out in a closed vessel, a maximum TON of 2,600 was measured after 24 h. During the catalysis, rhenium hydride and formate were observed as the two crucial intermediates. On the basis of experimental results, a straightforward two-step catalytic path was proposed; Re-H reacted with FA in the first step to produce the formate complex **18B**, which underwent decarboxylation to regenerate the hydride. The high-temperature stability of the catalyst and the production of CO-free H_2_ are the two major advantages of this method.

### 5.2 Manganese

**FIGURE 8:**
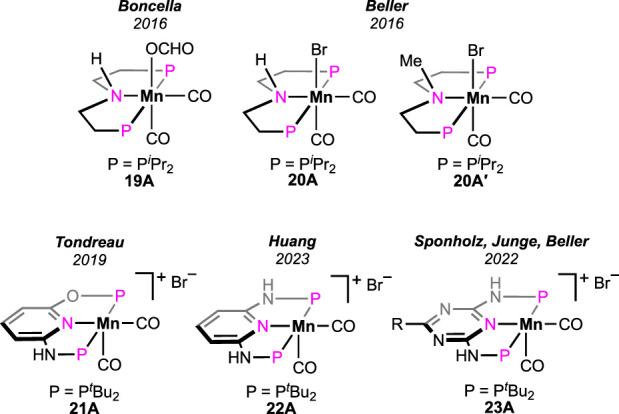
Manganese pincer catalysts for dehydrogenating formic acid.

In the literature, among diverse homogeneous catalysts, the application of manganese complexes has been extensively studied in recent times. Boncella et al. examined a manganese pincer complex **19A** (Figure 8), which is isoelectronic to catalyst **8A** (see Figure 5) (Tondreau and Boncella, 2016). Although positive activity in FA dehydrogenation was displayed by the catalyst, formation of CO and water were noticed from simultaneous FA dehydration. As noted during the process, these side products became responsible for catalyst decomposition. In contrast to this observation, only a negligible quantity of CO was formed in the case of iron catalyst **8A**. Additionally, for **8A**, an accelerating effect by Lewis acid was observed on the decarboxylation rate (Scheme 7, cycle 7b). In the case of **19A**, surprisingly, the conversion rate significantly slowed down after the use of LiBF_4_. In the same year, an analogous (PNP)-based manganese pincer with *P*-isopropyl substituents was reported by Beller et al. for methanol dehydrogenation (Figure 8, **20A**) (Anderez-Fernandez et al., 2016). The same catalyst, **20A**, was applied to FA decomposition, and it afforded carbon monoxide-free H_2_. Moderate turnovers were obtained in a propylene carbonate medium and in the presence of dimethyl octylamine. The proposed mechanism supported the participation of the manganese–nitrogen bond during catalysis, which differs from the (PNP)iron-catalyzed pathway (Scheme 7, cycle 7b). In a separate investigation, the group performed a reactivity comparison between **20A** and its N-methylated analog **20A′**. Poor catalytic activity was observed for **20A′**, suggesting an acceleration in the reaction rate through the cooperation of the central nitrogen in **20A**. A point to note is that in the case of similar ruthenium catalysts **5A** and **5A′**, surprisingly, the outcome was the opposite (see Figure 3; Scheme 5).

**SCHEME 13:**
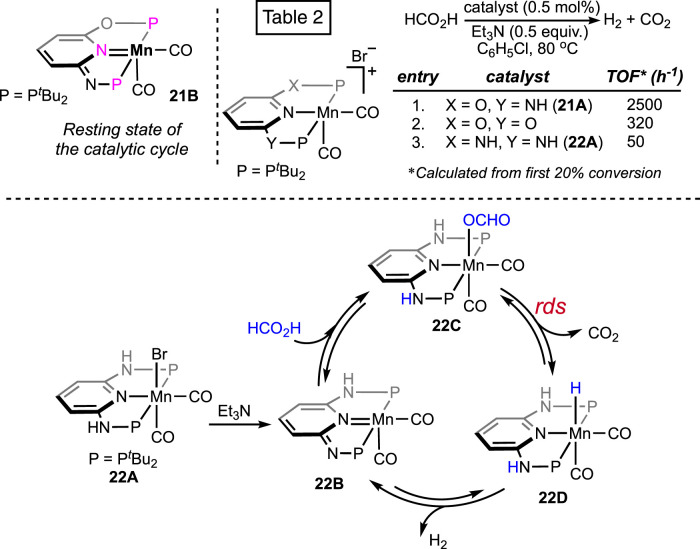
Proposed resting state of **21A**-catalyzed FAD and efficiency comparison of various manganese pincer complexes (above). Plausible base-free formic acid dehydrogenation pathway by the (PNNNP)Mn complex (below).

The catalytic efficiency of the manganese was hugely improved by applying the alternative (PNNOP)-ligand (Figure 8) (Anderson et al., 2019). Complex **21A** displayed a high TOF of 8,500 h^-1^ at 80°C. In the presence of bases such as triethyl and tributyl amine, a mixture of H_2_ and CO_2_ was produced in a closed vessel, building the pressure up to 18 atm. Furthermore, the catalyst maintained remarkable stability under the reaction condition even after 10 consecutive runs, projecting its rigidity and recyclability. Although mechanistic details were not described, complex **21B** was revealed as the resting state (Scheme 13), which strongly supported metal–ligand cooperativity. However, from the DFT study on **21A**-catalyzed FAD, Marino and Prejanò did not find a low-energy pathway involving ligand participation (Marino and Prejanò, 2021). Rather, a typical amine-assisted formation of Mn-OCHO, followed by the CO_2_ release without any backbone engagement, was found to be energetically favorable. Nevertheless, decarboxylation appeared as the *rds*, with the energy barrier determined to be 73.0 kJ mol^-1^. By comparison, structurally similar (^tBu^PONOP)- and (^tBu^PNNNP)-ligated complexes exhibited lower efficiency than **21A** (Scheme 13; Table 2).

Recently, the reactivity of the (PNNNP)-Mn pincer (**22A**) was explored by Huang et al. (Dutta et al., 2023). Although **22A** showed poor activity in chlorobenzene (Scheme 13; Table 2, entry 3), interestingly, when switching the solvent to DMSO, the TON increased to 274 at 80°C. With further escalation in temperature to 90°C, the turnover increased up to 15,200. A catalytic cycle involving ligand cooperativity was well established based on a series of experimental and spectroscopic data (Scheme 13). Complex **22B** was verified to be the entry point, which was generated through amine-assisted HBr elimination from the manganese bromide **22A**. Proposed intermediates **22B**–**D** were also detected in ^1^H NMR spectroscopy. The presence of reversibility throughout the whole catalytic cycle was further demonstrated. CO_2_ release was established to be the rate-determining step by the kinetic isotope effect studies.

**SCHEME 14:**
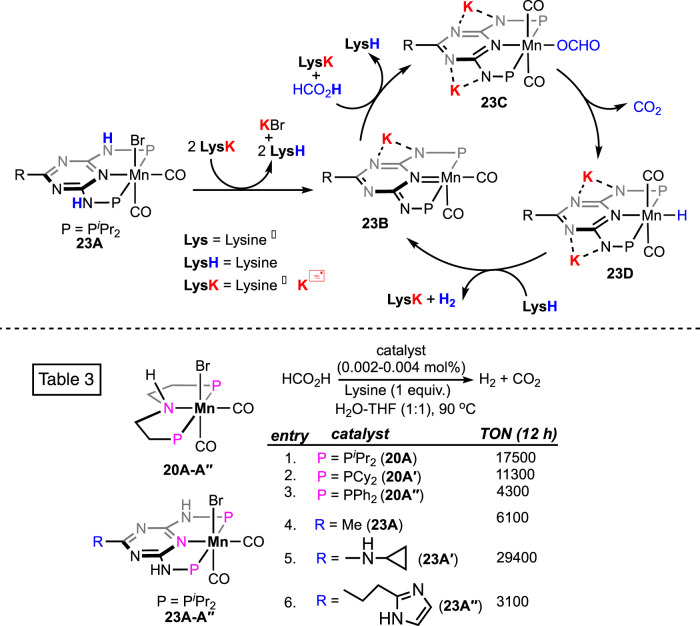
Proposed FA dehydrogenation pathway by (PN^Ar^NNP)Mn complexes in the presence of lysine as additive (above). Manganese pincer catalysts and their reactivity in formic acid dehydrogenation in the presence of lysine (below).

In 2022, extensive research on CO_2_ hydrogenation, along with FA dehydrogenation, was reported by Sponholz, Junge, and Beller using a new class of manganese pincer complexes (**23A**, see Figure 8) (Wei et al., 2022a; Wei et al., 2022b). The unique component in their method was the amino acid lysine, which was found to be essential for catalysis. At 90°C, under aqueous conditions (H_2_O:THF = 1:1), notably high turnovers were achieved in the presence of an equimolar amount of lysine. In a parallel study, after screening 14 amino acids, lysine and its salt were proven to be highly effective in trapping CO_2_ in an aqueous solution. Under the FA dehydrogenation condition, interestingly, the presence of a stoichiometric quantity of lysine successfully captured the produced CO_2_, which resulted in CO_2_-less H_2_ as the outcome. The ability to supply pure H_2_ (up to >99%) directly from the reaction mixture is another advantage of this procedure. Although both lysine (LysH) and its potassium salt (LysK) were found to be equally effective in facilitating catalysis, the latter is more potent in trapping CO_2_ and, hence, in generating pure H_2_ (>99%). A detailed mechanistic analysis demonstrated a lysine-mediated dehydrogenation pathway (Scheme 14). Mixing the pre-catalyst **23A** with LysK generated the active catalyst (**23B**). An NMR study supported the deprotonation of N-H on ligand arms by LysK. Using another molecule of LysK, the reaction between **23B** and FA produced the Mn–formate (**23C**), followed by its decarboxylation. In the next step, manganese hydride complex **23D** was protonated by lysine (LysH), forming H_2_ and regenerating the active catalyst. The substantial role of lysine and its salt in proton abstraction-donation throughout the cycle is quite explanatory for its essentiality in this process. In addition, an advantage of these catalysts was their reusability in H_2_ production from FA. In a follow-up study, under the same reaction condition, a reactivity comparison among various non-aromatic (PNP)-Mn pincers with varying *P*-substituents was carried out (Scheme 14; Table 3, entries 1–3), along with the effect of a distant ring substituent in catalysts **23A-Aʹʹ** (Scheme 14; Table 3, entries 4–6). The group also examined the H_2_ production from different formate salts (HCO_2_M; M^+^ = Li^+^, Na^+^, Cs^+^, NH_4_^+^, Mg^2+^, and Ca^2+^). All of them were found to behave similarly, and interestingly, glutamic acid was noted to be an effective CO_2_-trapping agent in addition to lysine.

## 6 Aluminum

**SCHEME 15:**
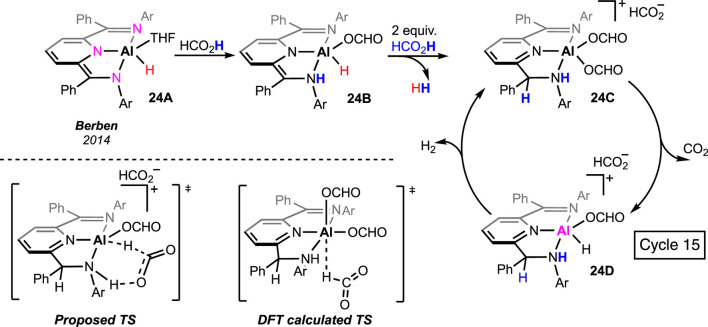
Formic acid dehydrogenation by aluminum pincer complexes.

Main group metal-based pincer complexes are rare in formic acid dehydrogenation. Berben and Myers prepared an aluminum hydride, **24A**, ligated with a pincer having three nitrogen donors (Myers and Berben, 2014; Lu et al., 2016). The complex offered decent reactivity in H_2_ production. In a stoichiometric reaction between the complex and formic acid, participation of the amide arm was observed (Scheme 15, **24A**→**24B**→**24C**). Moreover, double protonation of the ligand backbone was noted in the presence of three equivalent FAs, which was not observed for any other pincer complex. The diformate **24C** was believed to be the active catalyst, and a *β*-hydride elimination through a ligand-stabilized transition state was proposed by the group (Scheme 15, **24C**→**24D**). Fu et al. further studied this mechanism using DFT calculations. A non-hydrogen-bonded transition state was found to be associated with a lower energy barrier, suggesting no metal–ligand cooperativity during catalysis.

## 7 Challenges and future direction

Over the past decade, the discovery of pincer complexes played a pivotal role in advancing the catalytic FA dehydrogenation processes. More importantly, experimental observations along with theoretical calculation successfully provided a number of distinct catalytic pathways. Responses and outcomes from the variation in electronics and sterics around the active site offered a detailed understanding and the technicalities associated with the process. For instance, functional groups on the ligand backbone or the donor atoms have shown surprising effects on productivity. These insights tremendously helped to identify the existing challenges and their origins. Needless to say, through pincer systems, various strategies were successfully implied, including (i) incorporation of the internal base through ligand modification in numerous ways; (ii) use of Lewis acids, ionic liquids, and amino acids as effective additives; (iii) applying bimetallic frustrated Lewis pair complexes; and (iv) catalyst immobilization on solid surfaces. Although significant progress was made and important details were uncovered, there is still vast scope for improvement.

(1) Except for a few catalytic cycles, the release of CO_2_ from the metal–formate complex was found to be the rate-limiting step. Therefore, to achieve more efficient dehydrogenation, lowering the activation barrier through the stabilization of the corresponding transition states will be a rational choice. As an example, Schneider and Hazari et al. demonstrated the importance of Lewis acids in facilitating decarboxylation. To the best of our knowledge, fewer attempts have been made to examine CO_2_ elimination, and it needs further attention. In this regard, the isolation of various M-OCHO complexes and investigating their reactivity toward decarboxylation will be a strategic approach.
(2) The use of organic and costly solvents is another disadvantage of the existing methods. Most catalysts displayed greater efficiency in a highly polar medium. Nevertheless, protic solvents are proven to be ineffective, likely due to the solubility and stability issues of the catalysts. Keeping the objective of cost-effective and environment-friendly methodologies in mind, reactions in water, alcohol, or under solvent-less conditions will be desired. Among existing pincer catalysts, Ru-pincer **6A** is capable of dehydrogenating neat FA. Furthermore, in the case of immobilized Ir-pincer **12A**, cesium formate was found to be an effective alternative to conduct solvent-less FAD. Clearly, this shows the scope for improvement in managing catalyst-related solubility and stability issues. A straightforward viewpoint could be the introduction of ionic or polar functionality on the ligand backbones.
(3) Although plenty of catalysts have shown their capability in producing CO-free FA decomposition, to obtain pure H_2_, the separation of CO_2_ from the product gas mixture requires an additional step. In this respect, a noteworthy discovery was made by Junge and Beller. Following their method, a supply of carbon dioxide-free H_2_ directly from the reaction mixture was accomplished (pure up to >99%). Applying lysine and its salt to trap the carbon dioxide was the key. Undoubtedly, this strategy unfolded the importance of CO_2_-capturing materials and their exploration in formic acid dehydrogenation (Wei et al., 2022).

Along with homogeneous catalysts, a variety of heterogeneous systems have been successful in FAD (Pan et al., 2016; Agapova et al., 2019). In this domain, palladium is the most applied transition metal in addition to gold and silver. However, to the best of our knowledge, the use of palladium, gold, and silver complexes under homogeneous conditions is scarce in producing H_2_ from FA. The use of main-group metals is also rare. Therefore, exploring other metals and different ligand platforms needs to be continued, which eventually leads to novel and improved catalysts.
